# Absence of electron-transfer-associated changes in the time-dependent X-ray free-electron laser structures of the photosynthetic reaction center

**DOI:** 10.7554/eLife.88955

**Published:** 2023-10-05

**Authors:** Gai Nishikawa, Yu Sugo, Keisuke Saito, Hiroshi Ishikita

**Affiliations:** 1 Department of Applied Chemistry, The University of Tokyo Tokyo Japan; 2 Research Center for Advanced Science and Technology, The University of Tokyo Tokyo Japan; Goethe University Germany; Goethe University Germany

**Keywords:** X-ray free-electron laser structure, electron transfer dynamics, type II reaction center, redox potential, charge separation, chlorophyll, None

## Abstract

Using the X-ray free-electron laser (XFEL) structures of the photosynthetic reaction center from *Blastochloris viridis* that show light-induced time-dependent structural changes (Dods et al., (2021) Nature *589*, 310–314), we investigated time-dependent changes in the energetics of the electron-transfer pathway, considering the entire protein environment of the protein structures and titrating the redox-active sites in the presence of all fully equilibrated titratable residues. In the dark and charge separation intermediate structures, the calculated redox potential (*E*_m_) values for the accessory bacteriochlorophyll and bacteriopheophytin in the electron-transfer-active branch (B_L_ and H_L_) are higher than those in the electron-transfer-inactive branch (B_M_ and H_M_). However, the stabilization of the charge-separated [P_L_P_M_]^•+^H_L_^•^**^–^** state owing to protein reorganization is not clearly observed in the *E*_m_(H_L_) values in the charge-separated 5 ps ([P_L_P_M_]^•+^H_L_^•^**^–^** state) structure. Furthermore, the expected chlorin ring deformation upon formation of H_L_^•^**^–^** (saddling mode) is absent in the H_L_ geometry of the original 5 ps structure. These findings suggest that there is no clear link between the time-dependent structural changes and the electron-transfer events in the XFEL structures.

## Introduction

**Figure 1.**
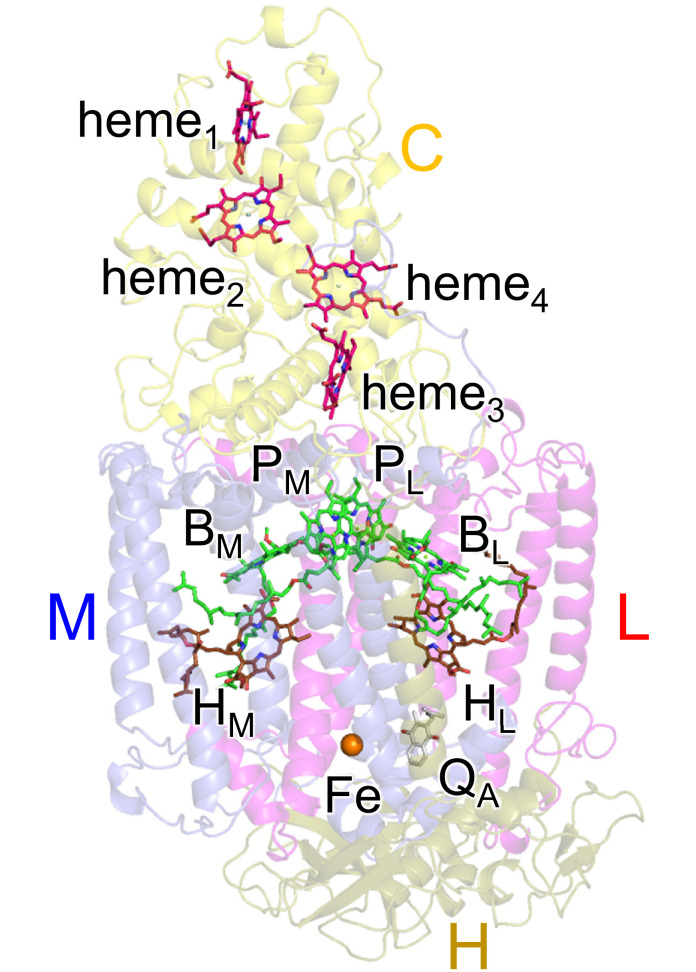
Electron-transfer pathways along the L- and M-branches in PbRC from *B. viridis*. The PbRC is composed of the L (red), M (blue), H (gold), and C (yellow) subunits. [P_L_P_M_]: BChl*b* pair; B_L_ and B_M_: accessory BChl*b*; H_L_ and H_M_: BPheo*b*; Q_A_: primary quinone (menaquinone); Fe: non-heme Fe complex.

Photosynthetic reaction centers from purple bacteria (PbRC) are heterodimeric reaction centers, which are formed by the protein subunits L and M (Figure 1). In PbRC from *Blastochloris viridis*, the electronic excitation of the bacteriochlorophyll *b* (BChl*b*) pair, [P_L_P_M_], leads to electron transfer to accessory BChl*b*, B_L_, followed by electron transfer via bacteriopheophytin *b* (BPheo*b*), H_L_, to menaquinone, Q_A_, along the electron-transfer active L-branch (A-branch) (Deisenhofer et al., 1985). Electron transfer further proceeds from Q_A_ to ubiquinone, Q_B_, which is coupled with proton transfer via charged and polar residues in the Q_B_ binding region (Rabenstein et al., 1998). Although the counterpart M-branch (B-branch) is essentially electron-transfer inactive, mutations of the Phe-L181/Tyr-M208 pair to tyrosine/phenylalanine lead to an increase in the yield of [P_L_P_M_]^•^**^+^**H_M_^•^**^–^** formation (~30%), which suggests that these residues are responsible for the energetic asymmetry in the electron-transfer branches (e.g., Kirmaier et al., 2003). The anionic states B_L_^•^**^–^**, H_L_^•^**^–^**, and Q_A_^•^**^–^** form in ~3.5 ps, ~5 ps, and ~200 ps upon the formation of the electronically excited [P_L_P_M_]* state, respectively (Holzapfel et al., 1990). The anionic state formation induces not only reoriganization of the protein environment (Marcus and Sutin, 1985) but also out-of-plane distortion of the chlorin ring (Saito et al., 2012). Two distinct conformations of H_L_^•^**^–^** were reported in spectroscopic studies of PbRC from *Rhodobacter sphaeroides* (Müh et al., 1998).

Recently, using the X-ray free-electron laser (XFEL), light-induced electron density changes and structural changes of PbRC were analyzed at 1 ps, 5 ps, 20 ps, 300 ps, and 8 μs upon the electronic excitation of [P_L_P_M_] at 960 nm (Dods et al., 2021): the 1 ps XFEL structure represents the [P_L_P_M_]* state, the 5 ps and 20 ps XFEL structures represent the charge-separated [P_L_P_M_]^•^**^+^**H_L_^•^**^–^** state, and the 300 ps and 8 μs XFEL structures represent the charge-separated [P_L_P_M_]^•^**^+^**Q_A_^•^**^–^** state. According to Dods et al., 2021, these XFEL structures revealed how the charge separation process was stabilized by protein conformational dynamics. However, the conclusions drawn from these XFEL structures are based on data with limited resolution. Specifically, eight out of nine XFEL structures have a relatively low resolution of 2.8 Å (atomic coordinates from PDB codes: 5O4C, 6ZI4, and 6ZI5 for dataset a and 6ZHW, 6ZID, 6ZI6, 6ZI9, and 6ZIA for dataset b) (Dods et al., 2021). In addition, the data statistics may indicate that the high-resolution range of some XFEL datasets exhibits high levels of noise (e.g., low CC_1/2_). These observations raise concerns about the reliable comparison of subtle conformational changes among these XFEL structures. Hence, caution must be exercised when interpreting these XFEL structures in terms of their ability to accurately capture relevant conformational changes.

Here, we investigated how the redox potential (*E*_m_) values of the BChl*b* and BPheo*b* cofactors for one-electron reduction change as electron transfer proceeds using the dark (0 ps), 1 ps, 5 ps, 20 ps, 300 ps, and 8 μs XFEL structures, solving the linear Poisson-Boltzmann equation, and considering the protonation states of all titratable sites in the entire protein. Structural changes (e.g., side-chain reorientation) in the protein environment can be analyzed in the *E*_m_ shift, as *E*_m_ is predominantly determined by the sum of the electrostatic interactions between the redox-active site and all other groups (i.e., residues and cofactors) in the protein structure. Subtle structural changes of the BChl*b* and BPheo*b* chlorin rings, which may not be pronounced even in the *E*_m_ shift (Saito et al., 2012), can be analyzed in the out-of-plane distortion of the chlorin rings using a normal-coordinate structural decomposition (NSD) analysis (Jentzen et al., 1997; Shelnutt et al., 1998) with a combination of a quantum mechanical/molecular mechanical (QM/MM) approach in the entire PbRC protein environment.

## Results and discussion

### Energetically asymmetric electron-transfer branches

**Figure 2.**
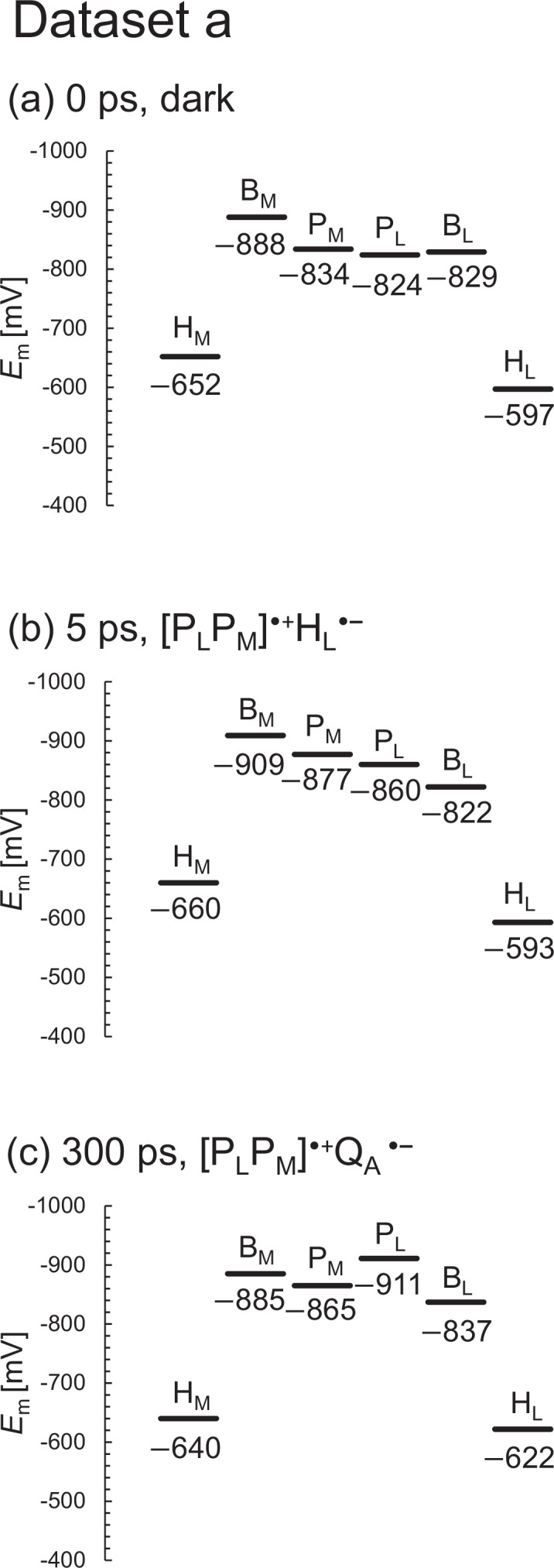
*E*_m_ profiles in the XFEL structures for dataset a. (**a**) 0 ps. (**b**) 5 ps. (**c**) 300 ps.

**Figure 3.**
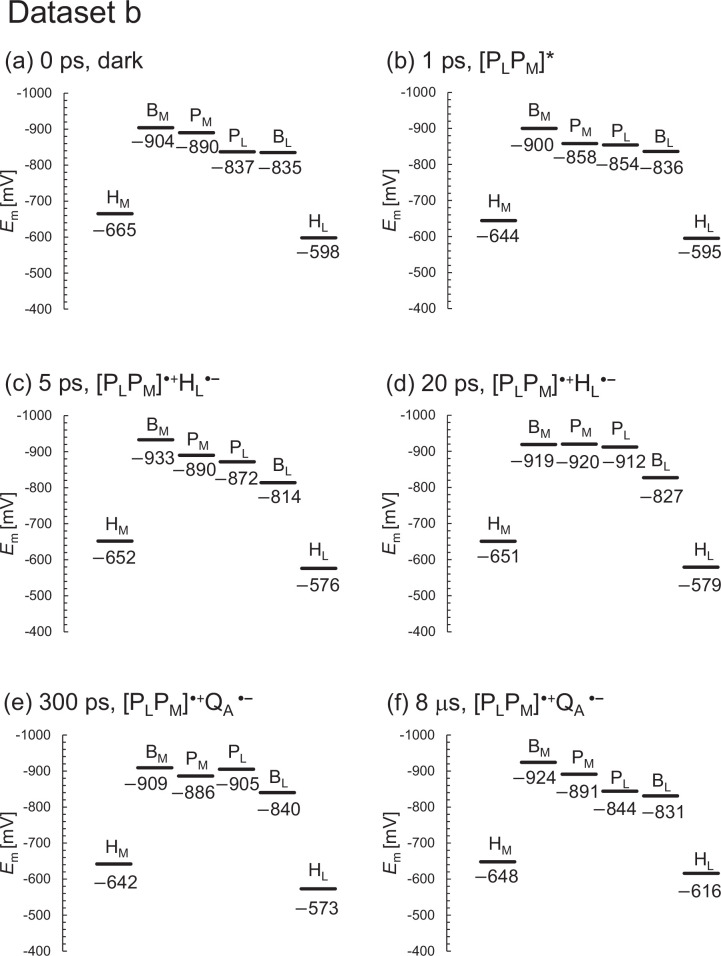
*E*_m_ profiles in the XFEL structures for dataset b. (**a**) 0 ps. (**b**) 1 ps. (**c**) 5 ps. (**d**) 20 ps. (**e**) 300 ps. (**f**) 8 μs.

The XFEL structures show that the *E*_m_ values for B_L_ are ~50 mV higher than those for B_M_, which facilitates the formation of the charge-separated [P_L_P_M_]^•+^B_L_^•^**^–^** state and thereby electron transfer along the L-branch (Figures 2 and 3). As the *E*_m_ profile is substantially consistent with the *E*_m_ profile for PbRC from *R. sphaeroides* (Kawashima and Ishikita, 2018), it seems plausible that the charge-separated [P_L_P_M_]^•+^B_L_^•^**^–^** and [P_L_P_M_]^•+^H_L_^•^**^–^** states in the active L-branch are energetically lower than the [P_L_P_M_]^•+^B_M_^•^**^–^** and [P_L_P_M_]^•+^H_M_^•^**^–^** states in the inactive M-branch, respectively, as demonstrated in QM/MM calculations (Tamura et al., 2020). Indeed, the calculated *E*_m_ values are largely correlated with the lowest unoccupied molecular orbital (LUMO) levels calculated using a QM/MM approach, as suggested previously (coefficient of determination *R*^2^=0.98, Figure 2—figure supplement 1). The *E*_m_(H_L_) value of –597 mV (in dataset a; –598 mV in dataset b) is in line with the experimentally estimated value of ca. –600 mV for H_L_ in PbRC from *B. viridis* (Rutherford et al., 1979).

**Figure 2—figure supplement 1.**
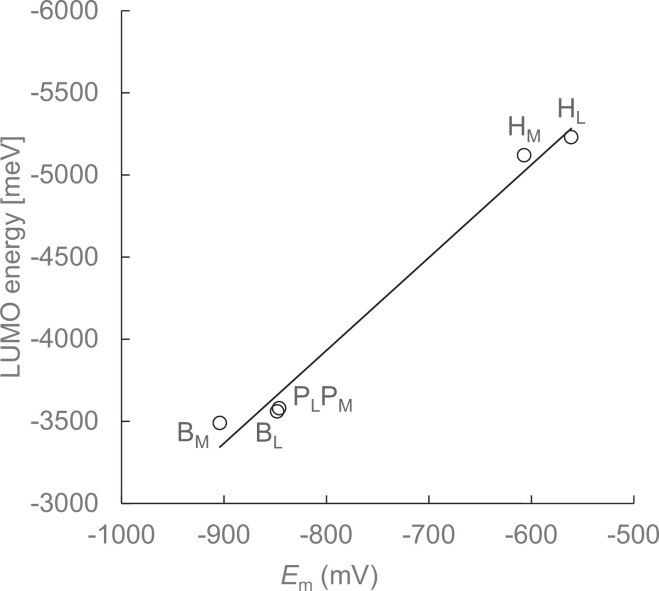
*E*_m_ values calculated solving the linear Poisson-Boltzmann equation and LUMO energy levels calculated using a QM/MM approach in the dark-state structure. Figure 2—figure supplement 1—source data 1.Numerical source data for Figure 2—figure supplement 1.

**Figure 4.**
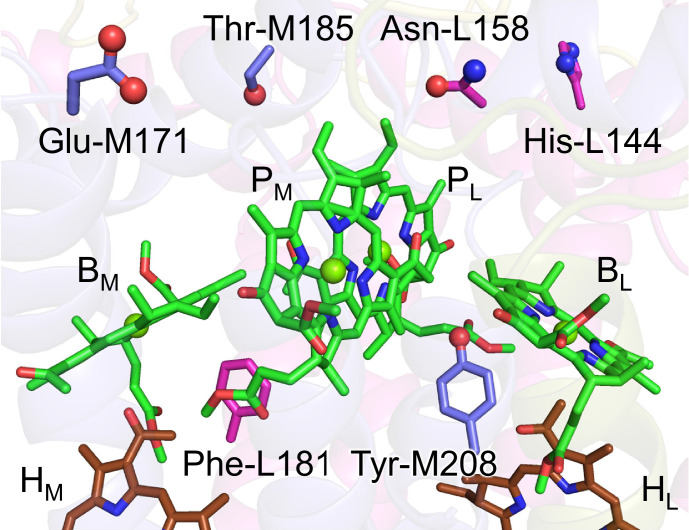
Residue pairs that are responsible for *E*_m_(B_L_)>*E*_m_(B_M_).

Among the L/M residue pairs, the Phe-L181/Tyr-M208 pair contributes to *E*_m_(B_L_)>*E*_m_(B_M_) most significantly (25 mV), facilitating L-branch electron transfer, as suggested in theoretical studies (Gunner et al., 1996; Table 1, Figure 2; Figure 3; Figure 4). This result is also consistent with the contribution of the Phe-L181/Tyr-M210 pair to the difference between *E*_m_(B_L_) and *E*_m_(B_M_), which was the largest in PbRC from *R. sphaeroides* (Parson et al., 1990) (26 mV; Kawashima and Ishikita, 2018). The Asn-L158/Thr-M185 pair also contributes to the difference between *E*_m_(B_L_) and *E*_m_(B_M_) (12 mV, Table 1), as does the Val-L157/Thr-M186 pair in PbRC from *R. sphaeroides* (22 mV; Kawashima and Ishikita, 2018).

**Table 1.** Contributions of the L/M residue pairs that are responsible for *E*_m_(B_L_)>*E*_m_(B_M_) (more than 10 mV) in the dark-state structure (mV). Difference: [contribution of subunit L to *E*_m_(B_L_)] + [contribution of subunit M to *E*_m_(B_L_)] – [contribution of subunit L to *E*_m_(B_M_)] – [contribution of subunit M to *E*_m_(B_M_)].

Subunit L	*E*_m_(B_L_)	*E*_m_(B_M_)	Subunit M	*E*_m_(B_L_)	*E*_m_(B_M_)	Difference
Phe-L181	0	17	Tyr-M208	39	–3	25
His-L144	–8	–2	Glu-M171	–14	–45	25
Asn-L158	5	–6	Thr-M185	–3	–4	12

For dataset b, the *E*_m_ values for H_L_ are >50 mV higher than those for H_M_ , as observed in *E*_m_(B_L_) and *E*_m_(B_M_) (Figure 3). However, the *E*_m_ difference decreases to ~30 mV in the 8 μs XFEL structure (Figure 3f), which implies that the 8 μs XFEL structure is distinct from the other XFEL structures (see below). Below, we discuss the dark-state structure if not otherwise specified.

**Figure 5.**
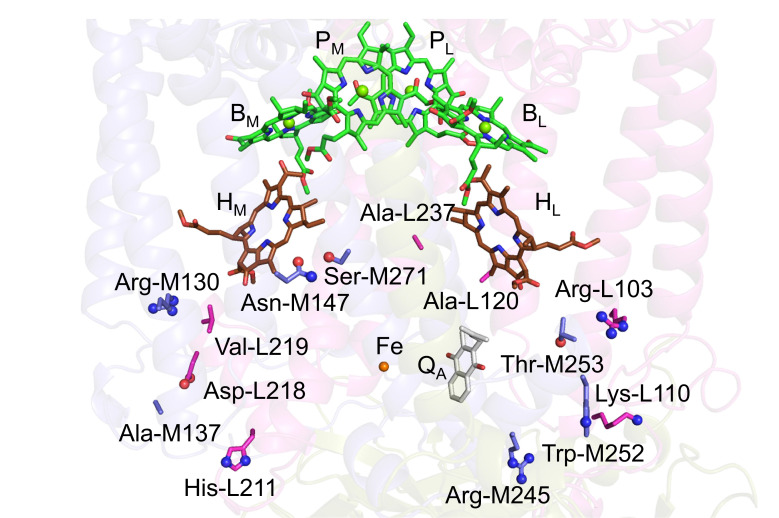
Residue pairs that are responsible for *E*_m_(H_L_)>*E*_m_(H_M_).

The Ala-L120/Asn-M147 pair contributes to *E*_m_(H_L_)>*E*_m_(H_M_) most significantly (38 mV) (Table 2, Figure 5). However, this holds true only for PbRC from *B. viridis*, as Asn-M147 is replaced with alanine (Ala-M149) in PbRC from *R. sphaeroides*. The Asp-L218/Trp-M252 pair decreases *E*_m_(H_M_) with respect to *E*_m_(H_L_), thereby contributing to *E*_m_(H_L_)>*E*_m_(H_M_) (20 mV) (Table 2; Figure 5). Arg-L103 orients toward the protein interior, whereas Arg-M130 orients toward the protein exterior (Figure 5), which contributes to *E*_m_(H_L_)>*E*_m_(H_M_) (17 mV) (Table 2). Ser-M271 forms an H-bond with Asn-M147 near H_M_ (Figure 5). Thus, the contribution of Ser-M271 to *E*_m_(H_L_) is large, although this residue is replaced with alanine (Ala-M273) in PbRC from *R. sphaeroides*.

**Table 2.** Contributions of the L/M residue pairs that are responsible for *E*_m_(H_L_)>*E*_m_(H_M_) (more than 10 mV) in the dark-state structure (mV). Difference: [contribution of subunit L to *E*_m_(H_L_)] + [contribution of subunit M to *E*_m_(H_L_)] – [contribution of subunit L to *E*_m_(H_M_)] – [contribution of subunit M to *E*_m_(H_M_)].

Subunit L	*E*_m_(H_L_)	*E*_m_(H_M_)	Subunit M	*E*_m_(H_L_)	*E*_m_(H_M_)	Difference
Ala-L120	–4	0	Asn-M147	0	–42	38
Asp-L218	–2	–22	Trp-M252	1	0	20
Arg-L103	77	3	Arg-M130	3	59	17
Ala-L237	–2	0	Ser-M271	3	–16	16
Lys-L110	17	2	Ala-M137	0	3	14
Val-L219	1	5	Thr-M253	17	1	11
His-L211	1	0	Arg-M245	14	4	11

### Relevance of structural changes observed in XFEL structures

**Figure 6.**
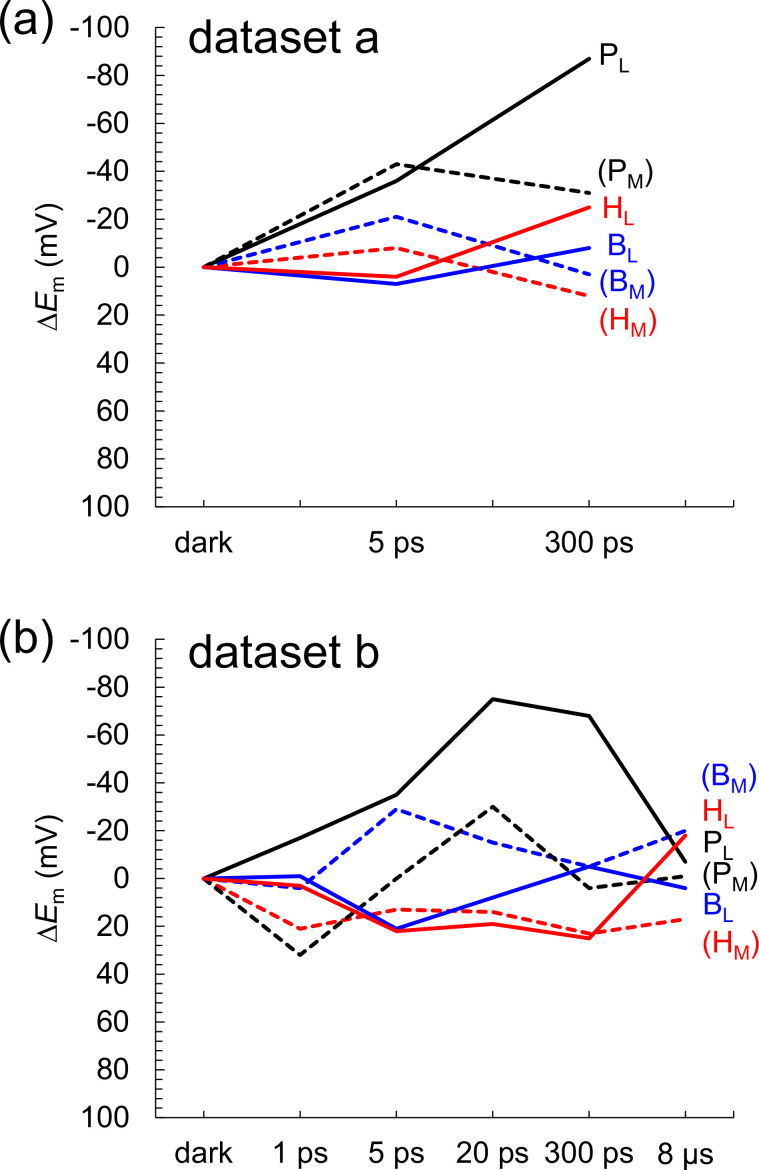
Time-dependent *E*_m_ changes for BChl*b* and BPheo*b* in the XFEL structures. (**a**) Dataset a. (**b**) Dataset b. Δ*E*_m_ denotes the *E*_m_ shift with respect to the dark-state structure. Black solid lines: P_L_; black dotted lines: P_M_; blue solid lines B_L_; blue dotted lines: B_M_; red solid lines: H_L_; red dotted lines: H_M_. Figure 6—source data 1.Numerical source data for Figure 6.

According to Dods et al., the 5 ps and 20 ps structures correspond to the charge-separated [P_L_P_M_]^•+^H_L_^•^**^–^** state (Dods et al., 2021). If this is the case, *E*_m_(H_L_) is expected to be exclusively higher in the 5 ps and 20 ps structures than in the other XFEL structures due to the stabilization of the [P_L_P_M_]^•+^H_L_^•^**^–^** state by protein reorganization. In dataset a, the *E*_m_(H_L_) value is only 4 mV higher in the 5 ps structure than in the dark structure (Figure 6a). In dataset b, the *E*_m_(H_L_) value is ~20 mV higher in the 5 ps and 20 ps structures than in the dark structure (Figure 6b). However, the *E*_m_(H_L_) value is 25 mV higher in the 300 ps structure than in the dark structure. Tables 3 and 4 show the residues that contribute to the slight increase in *E*_m_(H_L_) most significantly in the 5 ps and 20 ps structures. Most of these residues were in the region where Dods et al. specifically performed multiple rounds of partial occupancy refinement (e.g., 153–178, 190, 230, and 236–248 of subunit L and 193–221, 232, 243–253, 257–266 of subunit M) (Dods et al., 2021). In dataset b (Table 4), which has more data points than dataset a (Table 3), the contributions of these residues to *E*_m_(H_L_) often fluctuate (e.g., upshift/downshift followed by downshift/upshift) at different time intervals (e.g., 1–5 ps, 5–20 ps, and 20–300 ps). This result suggests that the structural differences among the XFEL structures are not related to the actual time course of charge separation. Furthermore, the *E*_m_(H_M_) value in the inactive M-branch is also ~15 mV higher in the 5 ps and 20 ps structures than in the dark structure (Figure 6b). These results suggest that the ~20 mV higher *E*_m_(H_L_) value in the 5 ps and 20 ps structures is not specifically due to the formation of the [P_L_P_M_]^•+^H_L_^•^**^–^** state. Thus, the stabilization of the [P_L_P_M_]^•+^H_L_^•^**^–^** state owing to protein reorganization is not clearly observed in the *E*_m_(H_L_) values.

**Table 3.** Residues that shift *E*_m_(H_L_) most significantly during putative electron transfer in the XFEL structures (dataset a) (mV). The same residues are highlighted in the same colors for clarity.

Dataset a		Shift		Shift
0–5 ps	Ser-L176	5	Cys-M210	4
	Thr-M220	–7	B_L_	–5
				
5–300 ps	B_L_	7	Gly-M209	3
	Gly-M211	–11	Leu-M212	–8

**Table 4.** Residues that shift *E*_m_(H_L_) most significantly during putative electron transfer in the XFEL structures (dataset b) (mV). The same residues are highlighted in the same colors for clarity.

Dataset b		Shift		Shift
0–1 ps	Ser-L238	8	Ser-L176	7
	B_L_	–7	Leu-M213	–3
				
1–5 ps	Gly-M211	6	Leu-M213	5
	Ser-L238	–6	Thr-M253	–5
				
5–20 ps	B_L_	12	Thr-M253	7
	Leu-M213	–4	P_M_	–3
				
20–300 ps	Ser-L238	3	Gly-M211	2
	B_L_	–10	Glu-L212	–4
				
300 ps to 8 μs	Glu-L212	4	Leu-M213	4
	B_L_	–6	Gly-M211	–5

**Figure 7.**
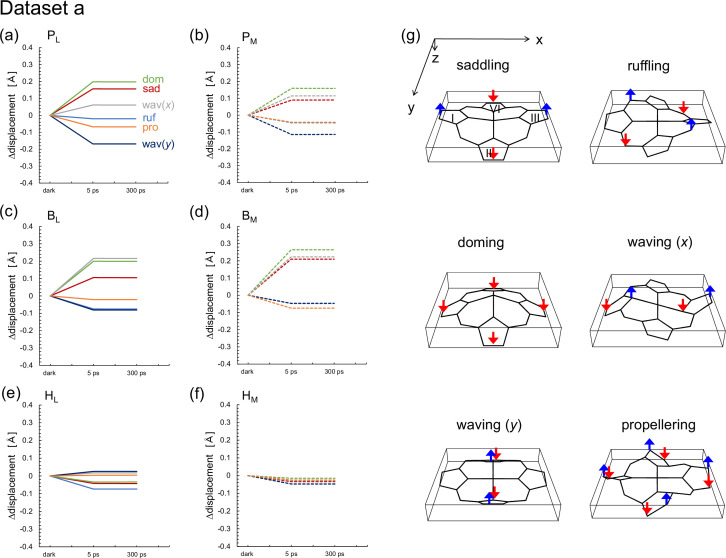
Time-dependent changes in the lowest frequency out-of-plane modes of the chlorin rings in the XFEL structures (dataset a). (a) P_L_. (b) P_M_. (c) B_L_. (d) B_M_. (e) H_L_. (f) H_M_. (g) Typical lowest frequency out-of-plane modes of the chlorin rings. Sad: saddling (red); ruf: ruffling (blue); dom: doming (green); wav(*x*, y): waving (x, y) (gray, dark blue); pro: propellering (orange). Solid and dotted lines indicate L- and M-branches, respectively. See Supplementary file 1 for the absolute values in the dark state for dataset a. Figure 7—source data 1.Numerical source data for Figure 7.

**Figure 8.**
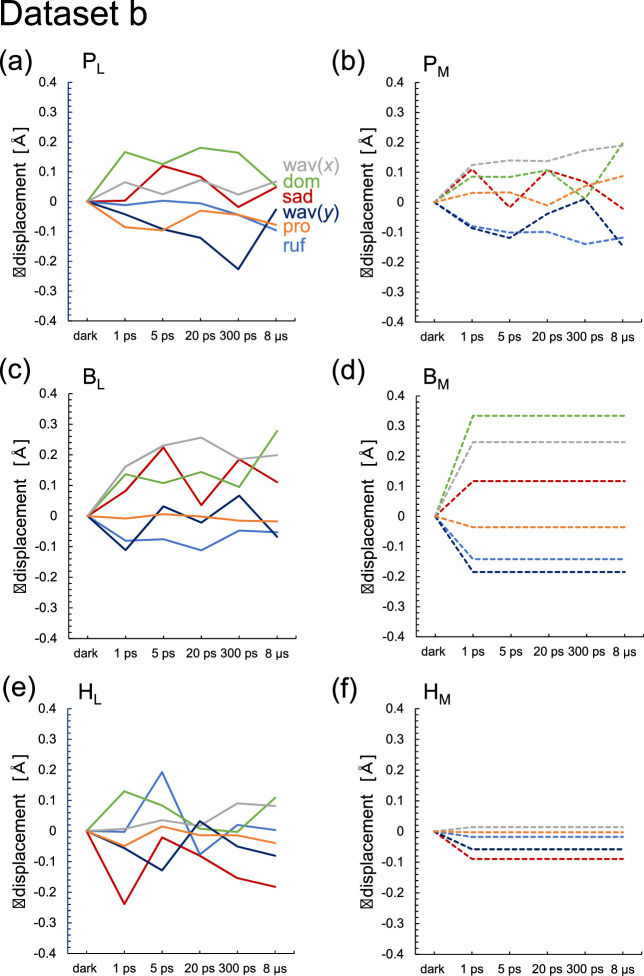
Time-dependent changes in the lowest frequency out-of-plane modes of the chlorin rings in the XFEL structures (dataset b). (a) P_L_. (b) P_M_. (c) B_L_. (d) B_M_. (e) H_L_. (f) H_M_. Sad: saddling (red); ruf: ruffling (blue); dom: doming (green); wav(*x*, y): waving (x, y) (gray, dark blue); pro: propellering (orange). Solid and dotted lines indicate L- and M-branches, respectively. See Supplementary file 2 for the absolute values in the dark state for dataset b Figure 8—source data 1. Figure 8—source data 1.Numerical source data for Figure 8.

An NSD analysis (Jentzen et al., 1997; Shelnutt et al., 1998) of the out-of-plane distortion of the chlorin ring is sensitive to subtle structural changes in the chlorin ring, which are not distinct in the *E*_m_ changes (Saito et al., 2012). QM/MM calculations indicate that H_L_^•^**^–^** formation induces the saddling mode in the chlorin ring, which describes the movement of rings I and III being in the opposite direction to the movement of rings II and IV along the normal axis of the chlorin ring (Tables 5 and 6). However, (i) in the XFEL structures, the saddling mode of H_L_ remains practically unchanged in dataset a during electron transfer (Figure 7 and Supplementary files 1 and 2). In dataset b, the saddling mode of H_L_ is induced most significantly at 1 ps, which does not correspond to the charge-separated [P_L_P_M_]^•+^H_L_^•^**^–^** state (Figure 8). (ii) In addition, the ruffling mode is more pronounced than the saddling mode in H_L_ (Figure 8), which suggests that the observed deformation of H_L_ is not directly associated with the reduction of H_L_.

**Table 5.** Induced out-of-plane distortion of H_L_ and H_M_ in the PbRC protein environment of the dark structure for dataset a in response to the reduction (Å). Table 5—source data 1.Numerical source data for Table 5.

	Saddling	Ruffling	Doming	Waving		Propellering
	B_2u_	B_1u_	A_2u_	E_g(x)_	E_g(y)_	A_1u_
H_L_	0.18	0.35	–0.10	0.13	–0.11	0.13
H_L_^•^**^–^**	0.24	0.35	–0.09	0.12	–0.12	0.13
(P_L_^•+^H_L_^•^**^–^**)	(0.22)	(0.36)	(–0.07)	(0.13)	(–0.13)	(0.13)
H_L_/H_L_^•^**^–^** difference	0.06	0.00	0.01	–0.01	–0.01	0.00
						
H_M_	0.06	0.40	–0.20	0.37	0.12	0.19
H_M_^•^**^–^**	0.12	0.38	–0.22	0.33	0.09	0.22
(P_L_^•+^H_M_^•^**^–^**)	(0.14)	(0.38)	(–0.22)	(0.33)	(0.10)	(0.22)
H_M_/H_M_^•^**^–^** difference	0.06	–0.02	–0.02	–0.04	–0.03	0.03

**Table 6.** Induced out-of-plane distortion of H_L_ and H_M_ in the PbRC protein environment of the dark structure for dataset b in response to the reduction (Å). Table 6—source data 1.Numerical source data for Table 6.

	Saddling	Ruffling	Doming	Waving		Propellering
	B_2u_	B_1u_	A_2u_	E_g(x)_	E_g(y)_	A_1u_
H_L_	0.13	0.35	–0.13	0.07	–0.09	0.20
H_L_^•^**^–^**	0.25	0.34	–0.02	0.12	–0.16	0.13
(P_L_^•+^H_L_^•^**^–^**)	(0.23)	(0.34)	(–0.03)	(0.12)	(–0.16)	(0.12)
H_L_/H_L_^•^**^–^** difference	0.12	–0.01	0.11	0.05	–0.07	–0.07
H_M_	0.08	0.57	–0.11	0.16	0.20	0.32
H_M_^•^**^–^**	0.16	0.36	–0.19	0.36	0.18	0.21
(P_L_^•+^H_M_^•^**^–^**)	(0.16)	(0.36)	(–0.20)	(0.36)	(0.18)	(0.21)
H_M_/H_M_^•^**^–^** difference	0.08	–0.21	–0.08	0.20	–0.02	–0.11

**Figure 9.**
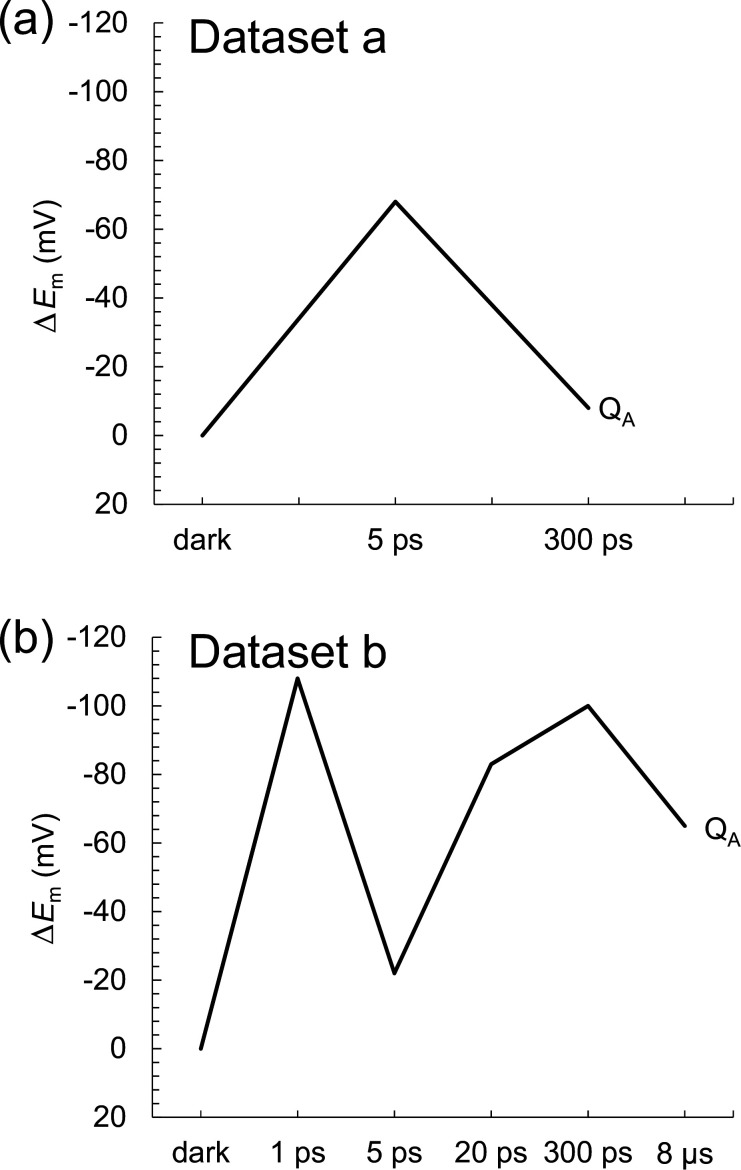
Time-dependent *E*_m_ changes for Q_A_ in the XFEL structures. (**a**) Dataset a. (**b**) Dataset b. Δ*E*_m_ denotes the *E*_m_ shift with respect to the dark-state structure. Note that the calculated *E*_m_(Q_A_) values for dataset a and dataset b in the dark structure are –223 mV and –209 mV, respectively, which are comparable to experimentally measured values of –150 mV for PbRC from *B. viridis* (menaquinone) (Prince et al., 1976) and –180 mV for PbRC from *R. sphaeroides* (ubiquinone) (Prince and Dutton, 1976). Figure 9—source data 1.Numerical source data for Figure 9.

One might argue that the loss of the link between the formation of the charge-separated state and the *E*_m_(H_L_) change (Figure 6) is not due to experimental errors, including data processing artifacts, but rather represents the actual ps timescale phenomena during the primary charge-separation reactions (e.g., Dods et al. noted that ‘the primary electron-transfer step to H_L_ is more rapid than conventional Marcus theory’; Dods et al., 2021). Even if this were the case, this hypothesis regarding the relevance of the XFEL structures to the electron-transfer events could be further explored by examining the changes in *E*_m_(Q_A_) among the XFEL structures, considering the relatively slow electron-transfer step to Q_A_ that allows sufficient protein relaxation to occur (e.g., Dods et al. stated that ‘the electron-transfer step to Q_A_ has a single exponential decay time of 230±30 ps, consistent with conventional Marcus theory’; Dods et al., 2021). That is, if the *E*_m_(Q_A_) values are not higher in the 300 ps and 8 μs structures than in the other structures, it suggests that significant experimental errors exist, rendering the XFEL structures irrelevant to the electron-transfer events. Consistent with this perspective, the present results demonstrate that the *E*_m_(Q_A_) values in the 300 ps and 8 μs structures are not significantly higher than those in the other structures, including the dark-state structure (Figure 9). Consequently, the lack of a clear relationship between the charge-separated state and the changes in *E*_m_(Q_A_) at 300 ps and 8 μs further strengthens the argument that the XFEL structures are irrelevant to the electron-transfer events.

In summary, the *E*_m_ values in the active L-branch are higher than those in the inactive M-branch in the XFEL structures, which suggests that electron transfer via B_L_^•^**^–^** and H_L_^•^**^–^** is energetically more favored than that via B_M_^•^**^–^** and H_M_^•^**^–^** (Figure 2). The Phe-L181/Tyr-M208 pair contributes to the difference between *E*_m_(B_L_) and *E*_m_(B_M_) the most significantly, as observed in the Phe-L181/Tyr-M210 pair in PbRC from *R. sphaeroides* (Kawashima and Ishikita, 2018; Parson et al., 1990). The stabilization of the [P_L_P_M_]^•+^H_L_^•^**^–^** state owing to protein reorganization is not clearly observed in the *E*_m_(H_L_) values (Figure 6). The absence of the induced saddling mode in the H_L_ chlorin ring in the 5 ps and 20 ps structures suggests that H_L_^•^**^–^** does not specifically exist in these XFEL structures (Figures 7 and 8). The cyclic fluctuations in the contributions of the residues to *E*_m_(H_L_) at different time intervals suggest that the structural differences among the XFEL structures are not related to the actual time course of charge separation (Table 4). The major limitation of the structural studies conducted by Dods et al., 2021, is the relatively low resolution of their XFEL structures, primarily at 2.8 Å. Consequently, the observed changes in *E*_m_ values and chlorin ring deformations are more likely to reflect experimental errors or data processing artifacts rather than actual structural changes induced by electron-transfer events. This concern is reinforced by the lack of a clear relationship between the actual Q_A_^•^**^–^** formation and the *E*_m_(Q_A_) values in the 300 ps and 8 μs structures (Figure 9). Consequently, the time-dependent structural changes proposed by Dods et al., 2021, are highly likely irrelevant to the electron-transfer events.

Hence, it is crucial to exercise caution when interpreting time-dependent XFEL structures, especially in the absence of comprehensive evaluations of the energetics for accompanying structural changes. This cautionary note should serve as a counterargument in the future, highlighting the potential pitfalls associated with presenting time-dependent XFEL structures of insufficient quality and drawing conclusive interpretations of protein structural changes that may not be distinguishable from significant experimental errors or data processing artifacts. Future high-resolution structures may provide further insights into the actual structural changes relevant to electron-transfer events. By combining both high-resolution structures and rigorous energetic evaluations, a more comprehensive understanding of the protein structure-function relationship can be achieved.

## Methods

### Coordinates and atomic partial charges

The atomic coordinates of PbRC from *B. viridis* were taken from the XFEL structures determined at 0 ps (dark state; PDB code 5O4C for dataset a and 5NJ4 for dataset b), 1 ps ([P_L_P_M_]* state; PDB code, 6ZHW for dataset b), 5 ps ([P_L_P_M_] ^•+^H_L_^•^**^–^** state; PDB code, 6ZI4 for dataset a and 6ZID for dataset b), 20 ps ([P_L_P_M_] ^•+^H_L_^•^**^–^** state; PDB code, 6ZI6 for dataset b), 300 ps ([P_L_P_M_] ^•+^Q_A_^•^**^–^** state; PDB code, 6ZI5 for dataset a and 6ZI9 for dataset b), and 8 μs ([P_L_P_M_] ^•+^Q_A_^•^**^–^** state; PDB code, 6ZIA for dataset b). Atoms with 30% occupancy for the photoactivated state (Dods et al., 2021) were used wherever present. Hydrogen atoms were generated and energetically optimized with CHARMM (Brooks et al., 1983). The atomic partial charges of the amino acids were obtained from the all-atom CHARMM22 (MacKerell et al., 1998) parameter set. For diacylglycerol, the Fe complex (Kawashima and Ishikita, 2018), and menaquinone (Kawashima and Ishikita, 2017), the atomic charges were adopted from previous studies. The atomic charges of BChl*b* and BPheo*b* (BChl*b*, BChl*b*^•+^, BChl*b*^•–^, BPheo*b*, and BPheo*b*^•–^) were determined by fitting the electrostatic potential in the neighborhood of these molecules using the RESP procedure (Bayly et al., 1993; Supplementary file 3). The electronic densities were calculated after geometry optimization using the density functional theory (DFT) method with the B3LYP functional and 6-31G** basis sets in the JAGUAR program (Jaguar, 2012). For the atomic charges of the nonpolar CH*_n_* groups in the cofactors (e.g., the phytol chains of BChl*b* and BPheo*b* and the isoprene side chains of quinone), a value of +0.09 was assigned to nonpolar H atoms.

### Calculation of *E*_m_: solving the linear Poisson-Boltzmann equation

The *E*_m_ values in the protein were determined by calculating the electrostatic energy difference between the two redox states in a reference model system. This was achieved by solving the linear Poisson-Boltzmann equation with the MEAD program (Bashford and Karplus, 1990) and using *E*_m_(BChl*b*) = –665 mV and *E*_m_(BPheo*b*) = –429 mV (based on *E*_m_(BChl*b*) = –700 mV and *E*_m_(BPheo*b*) = –500 mV for one-electron reduction measured in dimethylformamide; Fajer et al., 1976; Watanabe and Kobayashi, 1991), considering the solvation energy difference. The *E*_m_(Q_A_) value was calculated, using the reference *E*_m_ value of –256 mV versus NHE for menaquinone-2 in water (Kishi et al., 2017). The difference in the *E*_m_ value of the protein relative to the reference system was added to the known *E*_m_ value. To account for the ensemble of protonation patterns, a Monte Carlo method with Karlsberg was used for sampling (Rabenstein and Knapp, 2001). The linear Poisson-Boltzmann equation was solved using a three-step grid-focusing procedure with resolutions of 2.5 Å, 1.0 Å, and 0.3 Å. Monte Carlo sampling provided the probabilities [*A_ox_*] and [*A_red_*] of the two redox states of molecule *A*, and *E*_m_ was evaluated using the Nernst equation. A bias potential was applied to ensure an equal amount of both redox states ([*A_ox_*] = [*A_red_*]), thus determining the redox midpoint potential as the resulting bias potential. To ensure consistency with previous computational results, we used identical computational conditions and parameters as previous studies (e.g., Kawashima and Ishikita, 2018), performing all computations at 300 K, pH 7.0, and an ionic strength of 100 mM. The dielectric constants were set to 4 for the protein interior and 80 for water.

### QM/MM calculations

We employed the restricted DFT method for describing the closed-shell electronic structure and the unrestricted DFT method for the open-shell electronic structure with the B3LYP functional and LACVP* basis sets using the QSite (QSite, 2012) program. To neutralize the entire system, counter ions were added randomly around the protein using the Autoionize plugin in VMD (Humphrey et al., 1996). In the QM region, all atom positions were relaxed in the QM region, while the H-atom positions were relaxed in the MM region. The QM regions were defined as follows: for the BChl*b* pair [P_L_P_M_]: the side chains of the ligand residues (His-L173 and His-M200) and H-bond partners (His-L168, Tyr-M195, and Thr-L248); for accessory BChl*b*: B_L_/B_M_ and the side chain of the ligand residue (His-L153 for B_L_/His-M180 for B_M_); for BPheo*b*: H_L_/H_M_.

### NSD analysis

To analyze the out-of-plane distortions of chlorin rings, we employed an NSD procedure with the minimal basis approximation, where the deformation profile can be represented by the six lowest-frequency normal modes, that is, ruffling (B_1u_), saddling (B_2u_), doming (A_2u_), waving (E_g(x)_ and E_g(y)_), and propellering (A_1u_) modes (Jentzen et al., 1997; Shelnutt et al., 1998). The NSD analysis was performed in the following three steps, as performed previously (Saito et al., 2012). First, the atomic coordinates of the Mg-substituted macrocycle were extracted from the crystal or QM/MM optimized structures (Table 5—source data 1, Table 6—source data 1). Second, the extracted coordinates were superimposed on the reference coordinates of the macrocycle. The superimposition is based on a least-square method, and the mathematical procedure is described in Zucchelli et al., 2007. Finally, the out-of-plane distortion in the superimposed coordinates was decomposed into the six lowest-frequency normal modes by the projection to the reference normal mode coordinates as

(1) $$d^{\Gamma }=\sum_{i=1}^{N}\Delta z_{i}{\left(n_{z}^{\Gamma }\right)}_{i},$$

where $d^{\Gamma }$ represents the distortion component of the mode Γ (i.e., Γ = B_1u_, B_2u_, A_2u_, E_g(x)_, E_g(y)_, or A_1u_), $\Delta z_{i}$ is the *z*-component of the superimposed coordinates in the *i*th heavy atom, and ${\left(n_{z}^{\Gamma }\right)}_{i}$ is the *z*-component of the normalized eigenvector of the reference normal mode Γ in the *i*th heavy atom. *N* represents the number of heavy atoms. See Saito et al., 2012, for further details.

## Data Availability

Figure 2 - figure supplement 1 - source data 1, Figure 6 - source data 1, Figure 7 - source data 1, Figure 8 - source data 1, Figure 9 - source data 1, Table 5 - source data 1, and Table 6 - source data 1 contain the numerical data used to generate the figures and tables.

## Additional files

Supplementary file 1. Out-of-plane distortions in the PbRC protein environment of the dark structure for dataset a (Å).

Supplementary file 2. Out-of-plane distortions in the PbRC protein environment of the dark structure for dataset b (Å).

Supplementary file 3. Atomic charges of BChl*b* and BPheo*b*.

MDAR checklist

## References

[bib1] Bashford D, Karplus M (1990). p*K*_a_’s of ionizable groups in proteins: atomic detail from a continuum electrostatic model. Biochemistry.

[bib2] Bayly CI, Cieplak P, Cornell WD, Kollman PA (1993). A well-behaved electrostatic potential based method using charge restraints for deriving atomic charges: the RESP model. The Journal of Physical Chemistry.

[bib3] Brooks BR, Bruccoleri RE, Olafson BD, States DJ, Swaminathan S, Karplus M (1983). CHARMM: A program for macromolecular energy, minimization, and dynamics calculations. Journal of Computational Chemistry.

[bib4] Deisenhofer J, Epp O, Miki K, Huber R, Michel H (1985). Structure of the protein subunits in the photosynthetic reaction centre of *Rhodopseudomonas viridis* at 3Å resolution. Nature.

[bib5] Dods R, Båth P, Morozov D, Gagnér VA, Arnlund D, Luk HL, Kübel J, Maj M, Vallejos A, Wickstrand C, Bosman R, Beyerlein KR, Nelson G, Liang M, Milathianaki D, Robinson J, Harimoorthy R, Berntsen P, Malmerberg E, Johansson L, Andersson R, Carbajo S, Claesson E, Conrad CE, Dahl P, Hammarin G, Hunter MS, Li C, Lisova S, Royant A, Safari C, Sharma A, Williams GJ, Yefanov O, Westenhoff S, Davidsson J, DePonte DP, Boutet S, Barty A, Katona G, Groenhof G, Brändén G, Neutze R (2021). Ultrafast structural changes within a photosynthetic reaction centre. Nature.

[bib6] Fajer J, Davis MS, Brune DC, Spaulding LD, Forman A (1976). Chlorophyll radicals and primary events. Brookhaven symposia in biology.

[bib7] Gunner MR, Nicholls A, Honig B (1996). Electrostatic potentials in *Rhodopseudomonas viridis* reaction centers: implications for the driving force and directionality of electron transfer. The Journal of Physical Chemistry.

[bib8] Holzapfel W, Finkele U, Kaiser W, Oesterhelt D, Scheer H, Stilz HU, Zinth W (1990). Initial electron-transfer in the reaction center from *Rhodobacter sphaeroides*. PNAS.

[bib9] Humphrey W, Dalke A, Schulten K (1996). VMD: visual molecular dynamics. Journal of Molecular Graphics.

[bib10] Jaguar (2012). Version 7.9.

[bib11] Jentzen W, Song XZ, Shelnutt JA (1997). Structural characterization of synthetic and protein-bound porphyrins in terms of the lowest-frequency normal coordinates of the macrocycle. The Journal of Physical Chemistry B.

[bib12] Kawashima K, Ishikita H (2017). Structural factors that alter the redox potential of quinones in cyanobacterial and plant photosystem I. Biochemistry.

[bib13] Kawashima K, Ishikita H (2018). Energetic insights into two electron transfer pathways in light-driven energy-converting enzymes. Chemical Science.

[bib14] Kirmaier C, Laible PD, Hanson DK, Holten D (2003). B-side charge separation in bacterial photosynthetic reaction centers: nanosecond time scale electron transfer from H_B_^–^ to Q_B_. Biochemistry.

[bib15] Kishi S, Saito K, Kato Y, Ishikita H (2017). Redox potentials of ubiquinone, menaquinone, phylloquinone, and plastoquinone in aqueous solution. Photosynthesis Research.

[bib16] MacKerell AD, Bashford D, Bellott M, Dunbrack RL, Evanseck JD, Field MJ, Fischer S, Gao J, Guo H, Ha S, Joseph-McCarthy D, Kuchnir L, Kuczera K, Lau FT, Mattos C, Michnick S, Ngo T, Nguyen DT, Prodhom B, Reiher WE, Roux B, Schlenkrich M, Smith JC, Stote R, Straub J, Watanabe M, Wiórkiewicz-Kuczera J, Yin D, Karplus M (1998). All-atom empirical potential for molecular modeling and dynamics studies of proteins. The Journal of Physical Chemistry. B.

[bib17] Marcus RA, Sutin N (1985). Electron transfers in chemistry and biology. Biochimica et Biophysica Acta.

[bib18] Müh F, Williams JC, Allen JP, Lubitz W (1998). A conformational change of the photoactive bacteriopheophytin in reaction centers from *Rhodobacter sphaeroides*. Biochemistry.

[bib19] Parson WW, Chu ZT, Warshel A (1990). Electrostatic control of charge separation in bacterial photosynthesis. Biochimica et Biophysica Acta.

[bib20] Prince RC, Dutton PL (1976). The primary acceptor of bacterial photosynthesis: Its operating midpoint potential?. Archives of Biochemistry and Biophysics.

[bib21] Prince RC, Leigh JS, Dutton PL (1976). Thermodynamic properties of the reaction center of *Rhodopseudomonas viridis* in vivo measurement of the reaction center bacteriochlorophyll-primary acceptor intermediary electron carrier. Biochimica et Biophysica Acta.

[bib22] QSite (2012). Version 5.8.

[bib23] Rabenstein B, Ullmann GM, Knapp E-W (1998). Energetics of electron-transfer and protonation reactions of the quinones in the photosynthetic reaction center of *Rhodopseudomonas viridis*. Biochemistry.

[bib24] Rabenstein B, Knapp E-W (2001). Calculated pH-dependent population and protonation of carbon-monoxy-myoglobin conformers. Biophysical Journal.

[bib25] Rutherford AW, Heathcote P, Evans MCW (1979). Electron-paramagnetic-resonance measurements of the electron-transfer components of the reaction centre of *Rhodopseudomonas viridis*. Oxidation–reduction potentials and interactions of the electron acceptors. Biochemical Journal.

[bib26] Saito K, Umena Y, Kawakami K, Shen JR, Kamiya N, Ishikita H (2012). Deformation of chlorin rings in the photosystem II crystal structure. Biochemistry.

[bib27] Shelnutt JA, Song X-Z, Ma J-G, Jia S-L, Jentzen W, J. Medforth C (1998). Nonplanar porphyrins and their significance in proteins. Chemical Society Reviews.

[bib28] Tamura H, Saito K, Ishikita H (2020). Acquirement of water-splitting ability and alteration of the charge-separation mechanism in photosynthetic reaction centers. PNAS.

[bib29] Watanabe T, Kobayashi M, Scheer H (1991). Electrochemistry of Chlorophylls.

[bib30] Zucchelli G, Brogioli D, Casazza AP, Garlaschi FM, Jennings RC (2007). Chlorophyll ring deformation modulates Qy electronic energy in chlorophyll-protein complexes and generates spectral forms. Biophysical Journal.

