# Supplementary material for: Absence of electron-transfer-associated changes in the time-dependent X-ray free-electron laser structures of the photosynthetic reaction center
Source: eLife. 2023 Oct 5;12:RP88955. doi: 10.7554/eLife.88955 (PMC10554733; doi:10.7554/eLife.88955)
Supplement: Supplementary file 1. [file elife-88955-supp1.docx]

**Supplementary file 1.** Out-of-plane distortions in the PbRC protein environment of the dark structure for dataset a (Å).

|  | **saddling** | **ruffling** | **doming** | **waving** |  | **propellering** |
| --- | --- | --- | --- | --- | --- | --- |
|  | **B_2u_** | **B_1u_** | **A_2u_** | **E_g(x)_** | **E_g(y)_** | **A_1u_** |
| P_L_ | 0.10 | -0.63 | -0.06 | -0.01 | 0.12 | -0.15 |
| P_M_ | -0.02 | -0.94 | -0.08 | 0.01 | 0.26 | -0.28 |
| B_L_ | -0.13 | 0.16 | -0.07 | 0.20 | 0.10 | 0.10 |
| B_M_ | -0.18 | -0.03 | 0.10 | 0.13 | 0.04 | 0.13 |
| H_L_ | 0.07 | 0.28 | -0.05 | 0.11 | 0.12 | 0.18 |
| H_M_ | 0.02 | 0.69 | -0.04 | 0.14 | 0.22 | 0.37 |
